# Leucinostatin acts as a co-inducer for heat shock protein 70 in cultured canine retinal pigment epithelial cells

**DOI:** 10.1007/s12192-019-01066-z

**Published:** 2020-01-15

**Authors:** Qingkang Lyu, Irene S. Ludwig, Peter J. S. Kooten, Alice J. A. M. Sijts, Victor P. M. G. Rutten, Willem van Eden, Femke Broere

**Affiliations:** 1 Department of Infectious Diseases and Immunology, Faculty of Veterinary Medicine, Utrecht University, Yalelaan 1, Utrecht, The Netherlands; 2 Department of Veterinary Tropical Diseases, Faculty of Veterinary Science, Pretoria University, Pretoria, South Africa

**Keywords:** Retinal pigment epithelial cell, Leucinostatin, Heat shock protein 70, Canine

## Abstract

Dysregulation of retinal pigment epithelium (RPE) cells is the main cause of a variety of ocular diseases. Potentially heat shock proteins, by preventing molecular and cellular damage and modulating inflammatory disease, may exert a protective role in eye disease. In particular, the inducible form of heat shock protein 70 (Hsp70) is widely upregulated in inflamed tissues, and in vivo upregulation of Hsp70 expression by HSP co-inducing compounds has been shown to be a potential therapeutic strategy for inflammatory diseases. In order to gain further understanding of the potential protective effects of Hsp70 in RPE cells, we developed a method for isolation and culture of canine RPE cells. Identity of RPE cells was confirmed by detection of its specific marker, RPE65, in qPCR, flow cytometry, and immunocytochemistry analysis. The ability of RPE cells to express Hsp70 upon experimental induction of cell stress, by arsenite, was analyzed by flow cytometry. Finally, in search of a potential Hsp70 co-inducer, we investigated whether the compound leucinostatin could enhance Hsp70 expression in stressed RPE cells. Canine RPE cells were isolated and cultured successfully. Purity of cells that strongly expressed RPE65 was over 90%. Arsenite-induced stress led to a time- and dose-dependent increase in Hsp70 expression in canine RPE cells in vitro. In addition, leucinostatin, which enhanced heat shock factor-1-induced transcription from the heat shock promoter in DNAJB1-luc-O23 reporter cell line, also enhanced Hsp70 expression in arsenite-stressed RPE cells, in a dose-dependent fashion. These findings demonstrate that leucinostatin can boost Hsp70 expression in canine RPE cells, most likely by activating heat shock factor-1, suggesting that leucinostatin might be applied as a new co-inducer for Hsp70 expression.

## Introduction

The retinal pigment epithelium (RPE) is a single layer of polarized pigmented cells, in between the retina and the choroid, which originated from the neural ectoderm (Hartnett 2005). RPE cells play a critical role in the protection of retina function and vision of the eye: they nourish photoreceptors, absorb stray light, and engulf and degrade shed photoreceptor outer segments. As part of the blood-retina barrier, RPE cells also have a crucial role in maintaining the immune privilege of the eye, and in modulating local immune responses. Dysregulation and death of RPE cells is thought to contribute to a number of ocular disorders, such as age-related macular degeneration (AMD) (Heller and Martin 2014), diabetic retinopathy (Cai et al. 2000), and uveitis (Konda et al. 1994). Eye diseases detected in dogs, in veterinary clinics, are quite similar to those found in human patients. Therefore, diseased dogs may serve as an excellent model to study potential treatments, also for human diseases. So far, there are no effective strategies to cure these eye diseases, in neither humans nor dogs. Therefore, a search for novel and effective agents to modulate and regenerate RPE cell functionality is needed.

Heat shock protein 70 (HSPA1A, HSPA1B), which is a member of a major heat shock protein family, is highly conserved, and ubiquitously expressed during cell stress. Hsp70 has been implicated in T cell regulation of various chronic inflammatory diseases, including rheumatoid arthritis (Van Eden et al. 2005), colitis (Tanaka et al. 2007), neurodegenerative diseases (Van Noort 2008), and experimental autoimmune uveoretinitis (Kitamei et al. 2007), which makes the molecule a potential therapeutic target in chronic inflammations. Moreover, overexpression of Hsp70 was reported to play an essential role in regulation of apoptosis (Beere et al. 2000; Saleh et al. 2000) and protection against inflammation (Wang et al. 2017).

In clinical ophthalmology, retinal laser therapy such as laser photocoagulation and non-damaging retinal laser therapy (NRT) are widely used, to treat various retinal diseases. Studies on therapeutic mechanisms of retinal laser therapy showed that Hsp70 was substantially induced in laser target sites of ARPE cells (Inagaki et al. 2015) and the RPE layer of the rabbit model (Desmettre et al. 2001; Wang et al. 2016). Such upregulated Hsp70 may be involved in the improvement of the physiological function of RPE cells. Consequently, boosting of Hsp70 expression in RPE cells has been proposed as a possible therapeutic strategy in eye diseases. Currently, pharmacological co-induction of Hsp70 by food- or herb-derived compounds shows great promise in the battle against chronic inflammatory diseases, by enhancing the physiological Hsp70 response. It has been reported that HSP co-inducers, such as Bimoclomol, Geranylgeranylacetone (GGA), and celastrol, have a beneficial effect in the treatment of various inflammatory diseases by boosting HSP expression in experimental animal models, such as cerebrovascular disorders (ErdÖ and ErdÖ 1998), neurodegenerative diseases (Chow and Brown 2007), and uveitis (Kitamei et al. 2007).

Transcription of HSPs is regulated by the heat shock transcription factor 1 (HSF1). Under normal conditions, HSF1 is in an inactive state in the cytoplasm where it stays bound to Hsp70, Hsp90, and Hsp40. Upon stress, HSF1 undergoes nuclear translocation, binds to heat shock promoter elements, and induces transcription of heat shock genes (HSE). So far, compounds that increase HSP expression in canine RPE cells and the mechanisms involved have not been studied.

Aiming at a potential model for modulating HSP70 expression in dogs, as a target and model species, we successfully isolated and cultured canine RPE. We observed that arsenite-stress induced a time- and dose-dependent increase in Hsp70 expression in RPE cells in vitro, and that leucinostatin, an antimicrobial and antitumor antibiotic produced by the fungus Paecilomyces lilacinus, enhanced Hsp70 expression in arsenite-stressed cells in a dose-dependent fashion. In the present study, we thus identify a novel compound, leucinostatin, that can boost Hsp70 expression by activating heat shock factor-1.

## Materials and methods

### Animals

All dogs used in the experiments were owned by the Department of Clinical Science of Companion Animals of the Faculty of Veterinary Medicine, Utrecht University. The breeds of dogs included beagle and mongrel dogs. Eyes were isolated from healthy dogs, without any eye diseases, that were euthanized for unrelated purposes. This study was approved by the Utrecht University animal experiments committee (approval number: AVD115002016531).

### Dog RPE isolation and culture

To purify primary RPE cells from dogs, eyes were taken out and placed in DMEM medium with penicillin-streptomycin and 10% bovine serum, and connective tissue was removed. Then, eyes were washed successively with 70% ethanol, penicillin-streptomycin, and PBS. Afterwards, the anterior segment of the eye was removed along the ora serrata. Vitreous in the eye cup was pushed out as much as possible and the eye cup was filled with 2 ml of PBS with 1 mM EDTA (pre-warmed to 37 °C), placed in a well of a 12-well tissue culture plate, and put in the incubator (37 °C and 5% CO_2_) for 20–30 min, to loosen the neural retina. Then the retina was peeled off gently and the eye cup was refilled with 3 ml of pre-warmed 0.05% trypsin (Gibco), and incubated for another 30–40 min at 37 °C. RPE cells were harvested by gentle pipetting. Cells were seeded in T-75 tissue culture flasks and expanded at 37 °C with 5% CO2 in DMEM (Gibco, Cat. No.: 31966021) containing 10% FBS, 100 units/mL penicillin and 100 μg/ml streptomycin, non-essential amino acids, and 1 ng/mL epidermal growth factor (SIGMA-ALDRICH, Cat. No.: E417). Cells were passaged at 80% confluency.

### The DNAJB1-Luc-O23 reporter cell line and luciferase assays

The DNAJB1-Luc-O23 cell line, a generous gift of Kampinga HH (Kampinga et al. 2009; Wieten et al. 2010a), was grown in DMEM supplemented with 10% FBS, 100 units/mL penicillin and 100 μg/mL streptomycin, and 1 mg/mL hygromycin (Roche Diagnostics GmbH), in an 37 °C incubator with 5% CO_2_.

After trypsinization, DNAJB-Luc-O23 cells were seeded into the wells of a white μClear 96-well plate (Greiner Bio-one, 1.5 × 10^4^ cells/well), and placed in DMEM medium with 10% FBS, 100 U/ml penicillin, and 100 μg/ml streptomycin. After 24 h, leucinostatin and sodium arsenite were added at specified concentrations (Fig. 7), and after o/n incubation, luciferase activity was measured with a Promega Steady-Glo Luciferase Assay System using a LB960 Microplate Luminometer (Berthold Technologies), according to the manufacturer’s instruction.

### Quantitative real time PCR

RPE cells were harvested at passages 1, 2, 3, and 4. Total RNA was isolated using an RNeasy kit (Qiagen, Venlo, the Netherlands), according to the manufacturer’s instructions, followed by DNase treatment. RNA concentrations were measured using a Nano-drop-1000 spectrophotometer, and mRNA was reverse-transcribed to cDNA using an iScript™ cDNA Synthesis Kit (Bio-Rad) and Bio-Rad Thermal Cycler, according to the manufacturer’s instructions.

Primers for RPE65 were synthesized by Invitrogen (Forward primer: 5′-GCCTCGTCAAGCCTTTGAGT-3′. Reverse primer: 5′-CTGATGGGTATGAGTCGGGC-3′). Quantitative PCR to detect RPE65 expression in RPE cells was performed using iQ™ SYBR Green Supermix (Bio-Rad) and 0.4 μM of RPE65 primers, applying the following cycle parameters: 3 min at 95 °C, followed by 40 cycles of 20 s at 95 °C and 45 s at 60 °C (Bio-Rad CFX Connect real time system). Relative expression of mRNA was calculated by the Pfaffl-method using the housekeeping gene RPS19, encoding the Ribosomal Protein S19, as a reference (Forward primer: 5′-CCTTCCTCAAAAAGTCTGGG-3′ Reverse primer: 5′-GTTCTCATCGTAGGGAGCAAG-3′).

### Hsp70 induction in RPE cells

RPE cells were seeded in 12-well plates (2–4 × 10^5^ cells/well) and cultured at 37 °C o/n. At the second day, the medium was refreshed, and cells were incubated with carvacrol (SIGMA-ALDRICH, Cat. No. 499-75-2) or leucinostatin (SIGMA-ALDRICH, Cat. No: SML1566) dissolved in vehicle (ethanol and DMSO respectively) at the concentrations indicated in the figures (Figs. 4, 5, 6, and 7). Control cultures were incubated with medium or vehicle alone. After 2 h, sodium arsenite was added to the cultures, at the concentrations indicated in Figs. 4, 5, 6, and 7. Cells were collected after time intervals of 4, 8, 16, or 32 h, and Hsp70 expression was analyzed as described below.

### Flow cytometric analysis of Hsp70 and RPE 65 expression

For analysis of intracellular Hsp70 expression, cells were fixed and permeabilized for 30 min in Cytofix/Cytoperm solution (BD Pharmingen), washed, and then incubated with either a fluorescein isothiocyanate–labeled monoclonal antibody (SPA-810; Stressgen) to specifically detect inducible Hsp70 (HSPA1A/HSPA1B), or with the corresponding isotype control antibody, in Perm/Wash (BD Pharmingen) supplemented with 2% normal mouse serum. For analysis of intracellular RPE65 expression, cells were collected at passages 1, 2, or 3, and fixed and permeabilized as above. Then RPE cells were stained with an RPE65 monoclonal antibody for 30 min. Afterwards, cells were washed two times and incubated WITH Goat anti-mouse IgG(H+L)-PE. For final analysis of fluorescence, a FACS Canto (BD Pharmingen) flow cytometer was used.

### Immunocytochemistry

Cells (1 × 10^5^ cells/well) were grown on Lab-Tek II chamber slides overnight to let them attach, and then fixed in 4% paraformaldehyde (PFA) in PBS for 10 min at room temperature. After that, cells were washed three times with ice-cold PBS and permeabilized with Perm/Wash (BD Pharmingen) for 30 min. Subsequently, cells were blocked with 3% dog serum in prewash at room temperature for 60 min. Then cells were incubated with anti-mouse RPE65 (1:100, Invitrogen, Cat. No. MA1-16578) overnight at 4 °C. Cells were washed three times, 5 min each, with Perm/Wash to remove unbound antibodies and incubated for 30 min at room temperature with anti-mouse AlexaFluor 488 (life technologies, Cat. No. A21121) 1:400 diluted in Perm/Wash. Afterwards, cells were washed three times and mounted with 40,60-diamino-2-phenylindole (DAPI) mounting medium to stain the cell nuclei. Finally, cells were imaged with a fluorescence microscope.

### Statistical analysis

GraphPad Prism 7.04 (GraphPad Software, La Jolla, CA, USA) was used for statistical analyses and graphical display of the data. For multiple comparisons, one-way or two-way ANOVA tests with Bonferroni correction were used. *P* values below 0.05 were considered statistically significant.

## Results

### Characterization of cultured dog RPE cells

**Fig. 1:**
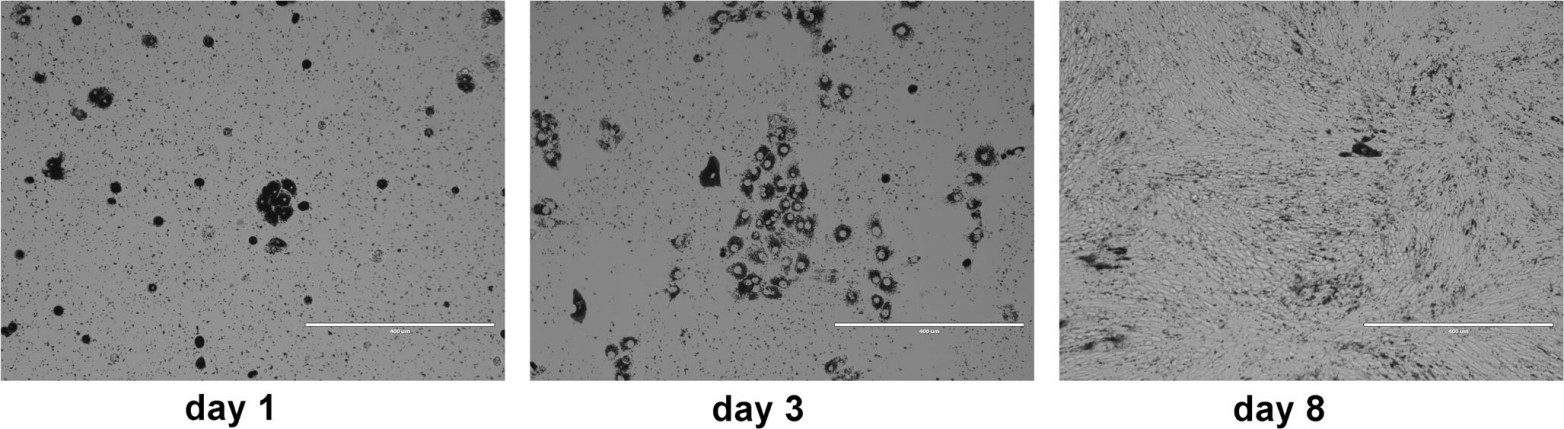
Isolation of primary RPE cells from dog eyes. Light micrographs of RPE cells at 1, 3, and 8 days after plating. Scale bar: 400 μm

Although most studies describe the isolation of RPE cells by peeling the RPE sheet from the choroid (Amirpour et al. 2014; Heller et al. 2015), it has proven to be challenging to obtain pure populations of RPE cells, due to firm attachment to the Bruch’s membrane and choroid. Therefore, we developed a method that does not rely on peeling off the RPE layer. To this end, the eye anterior segment and neural retina were removed, and then trypsin was added directly into the remaining posterior eye cup to digest the RPE layer, as described in “Materials and methods.” A few hours after seeding into the flasks, most of the RPE cells had attached (Fig. 1, day 1). Primary cultures of dog RPE cells grew as hexagonal, pigmented cells, and reached confluency after 8 days of culture. However, RPE cell pigmentation reduced with the number of cell divisions (Fig. 1, day 3 and day 8).

**Fig. 2:**
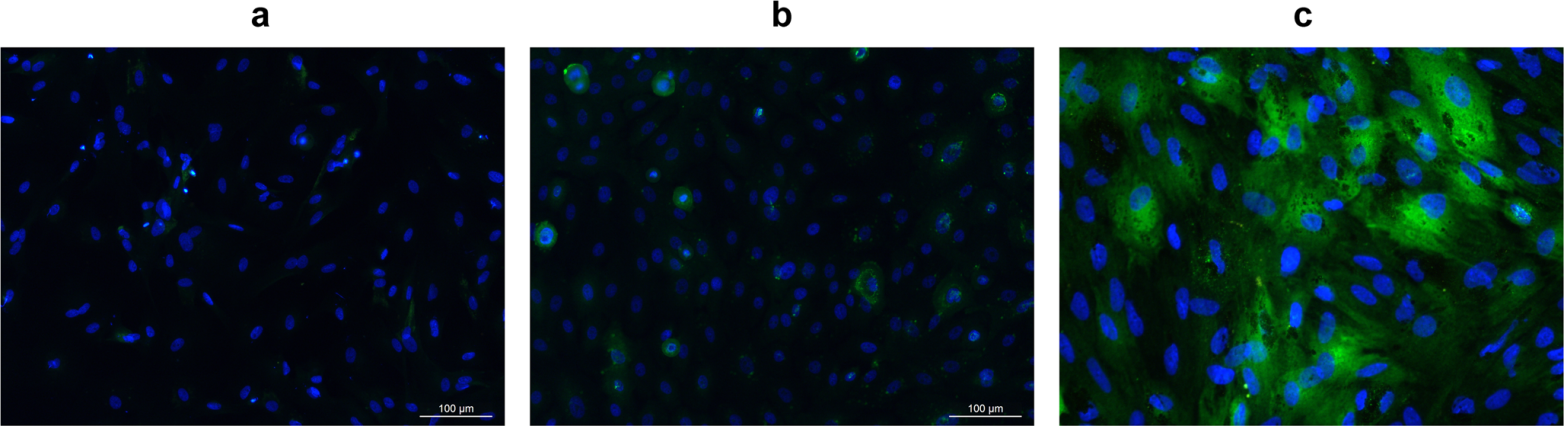
Expression of RPE65 protein in cultured dog RPE cells. Cells (in passage 1) were fixed and stained with RPE65 antibody to label RPE65 protein (green) and DAPI to label nuclei (blue). **a** DAPI was used to visualize cell nuclei in RPE cells; **b** RPE65 expression in canine epidermal keratinocytes (MSECK), as control group; **c** RPE65 expression in dog primary RPE cells

To test the RPE cell cultures for expression of the RPE cell-specific marker RPE65, primary cells in passage 1 were harvested and allowed to adhere to a coverslip. Then, cells were fixed and stained for immunofluorescence analysis. As shown in Fig. 2c, over 90% of canine RPE cells expressed the cytoplasmic RPE65 protein, in contrast to control, primary canine fibroblasts (Fig. 2b).

**Fig. 3:**
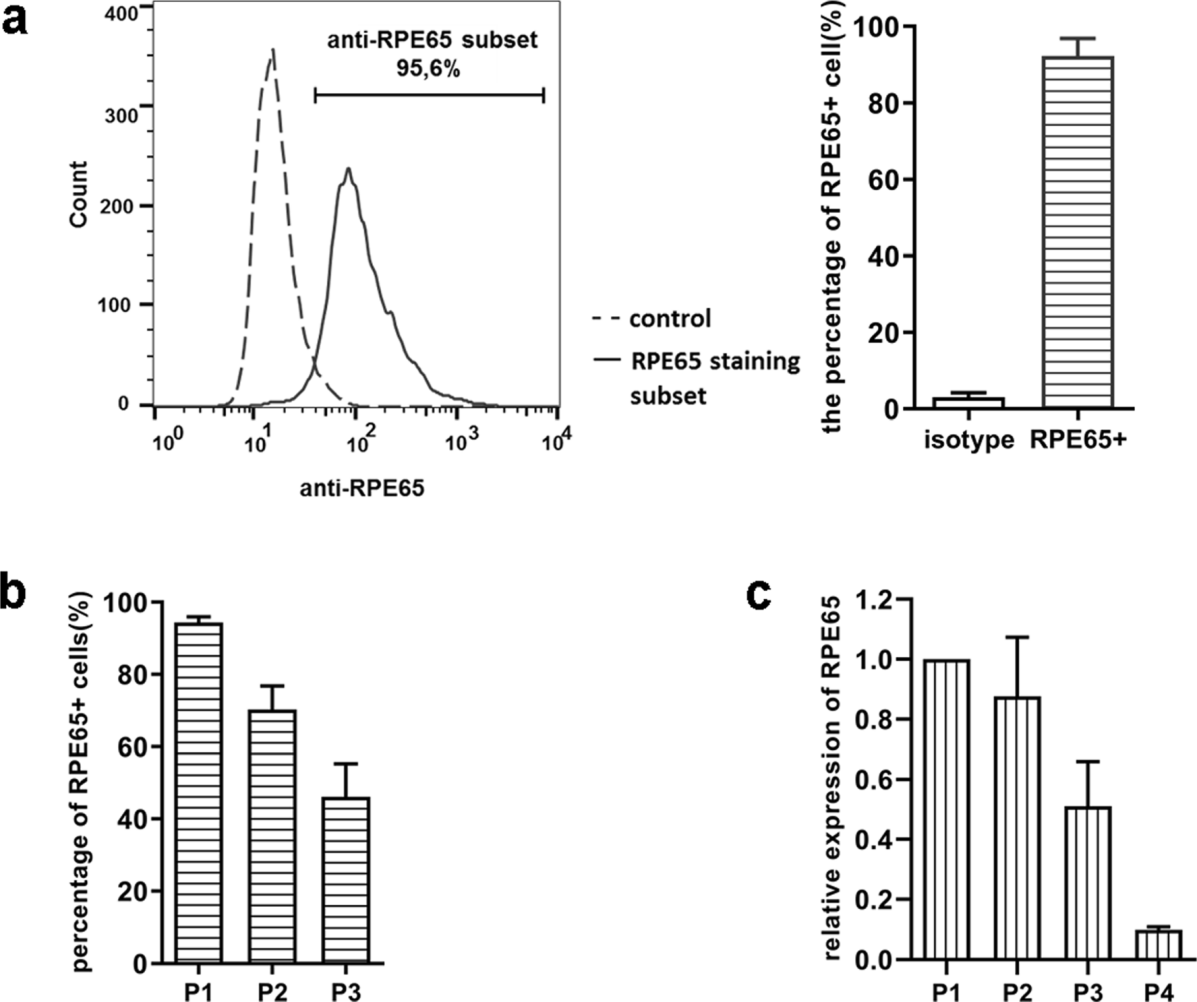
RPE65 protein expression analysis in different passages of dog primary RPE cells. RPE cells were stained with anti-RPE65 MoAb and analyzed by flow cytometry. **a** representative histogram (left) of the percentage of RPE65 expressing cells in dog RPE cell cultures in passage 1; the bar graph (right) shows the mean of three independent experiments; **b** percentage of RPE65 protein expressing cells in passages 1, 2, and 3; **c** gene expression comparison among different passages of cultured RPE cells (passages 1, 2, 3, and 4). RPE65 expression in passage 1 was regarded as maximum expression. Representative results from three separate experiments

To assess RPE65 expression in dog primary RPE cells, at different passages, we collected cells at passages 1, 2, 3, and 4 and measured RPE65 expression at protein and mRNA levels (Fig. 3). The percentage of RPE65 positive cells detected by flow cytometry (Fig. 3a, b) was approximately 95% in passage 1, and then decreased with increasing number of cell passage, which was in line with RPE65 mRNA expression in the different passages (Fig. 3c).

Hence, it was shown that the alternative RPE cell isolation method employed yielded a highly homogenous population of canine RPE cells, expressing RPE65 protein in passage 1. RPE65 expression however was gradually lost over time of culture, to approximately 10% of initial expression level in passage 4.

### Hsp70 production is induced in arsenite-stressed canine RPE cells

**Fig. 4:**
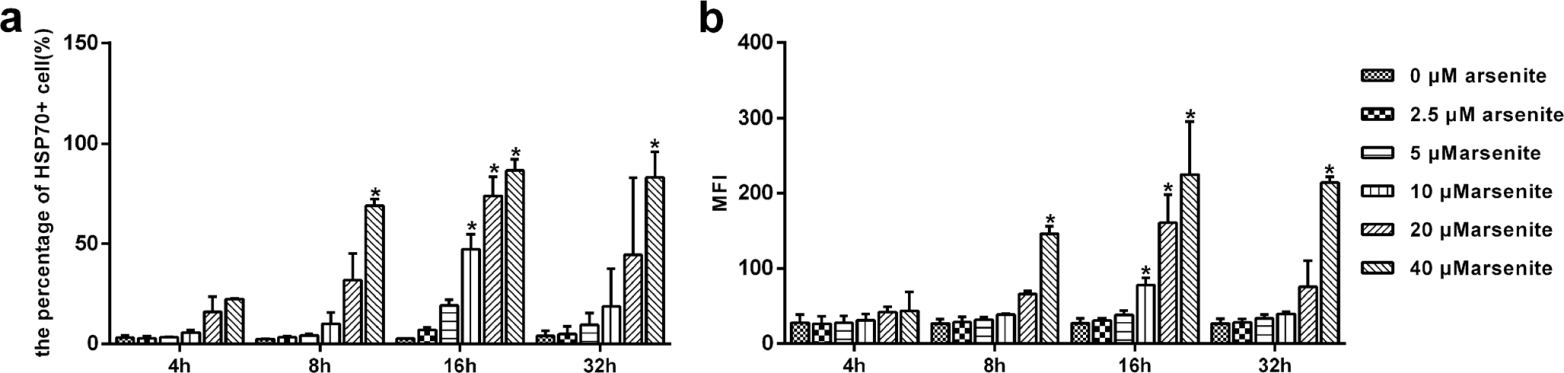
Sodium arsenite stimulation induces production of Hsp70 in dog primary RPE cells. RPE cells were incubated with sodium arsenite at the indicated concentrations, for 4, 8, 16, and 32 h at 37 °C. (a) Percentage of Hsp70 positive cells, detected by immunofluorescence staining with a MoAb, specific for inducible Hsp70, and flow cytometry. (b) Mean fluorescence intensity (MFI) of Hsp70+ cells. Data are representative of three independent experiments. *P* < 0.05 was considered statistically significant

To study whether stress upregulated Hsp70 production in RPE cells, the well-known stressor arsenite was chosen. Primary canine RPE cells were exposed to different concentrations of arsenite (0, 2.5, 5, 10, 20, and 40 μM) for 4, 8, 16, and 32 h, and Hsp70 protein expression was measured by flow cytometry (Fig. 4). We found that arsenite when applied at concentrations ranging from 5 to 40 μM induced a dose-dependent increase in Hsp70 expression. The most significant differences were observed following a 16-h incubation, at different arsenite concentrations. These data indicate that arsenite-stress induces a time- and dose-dependent increase in expression of Hsp70 in cultured canine RPE cells.

### Effect of carvacrol and leucinostatin on the induction of Hsp70 in stressed canine RPE cells

**Fig. 5:**
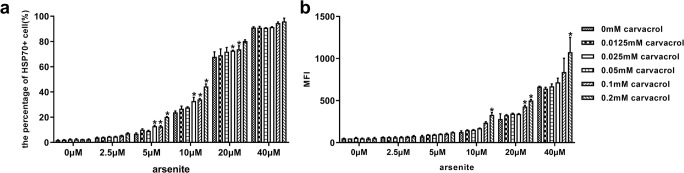
Carvacrol co-induced Hsp70 expression in dog primary RPE cell (passages 2–4). RPE cells were incubated with the indicated concentration of carvacrol for 2 h at 37 °C, followed by incubation with sodium arsenite at the indicated concentrations. After 16 h, Hsp70 expression in RPE cells was detected by immunofluorescence staining and flow cytometry. (a) Mean percentages of sodium arsenite- and/or carvacrol-treated RPE cells, expressing Hsp70; (b) mean fluorescence intensity (MFI) of Hsp70 expression, detected in treated, cultured RPE cells.. Data are representative of three independent experiments. *P* < 0.05 was considered statistically significant

To determine whether Hsp70 could be co-induced in canine RPE cells, carvacrol, known to co-induce Hsp70 in various cell types (Burt et al. 2007; Wieten et al. 2010b), was used. RPE cells were incubated for 16 h with arsenite and the indicated concentrations of carvacrol (Fig. 5). We found that carvacrol upregulated Hsp70 expression dose-dependently in cells stressed by 5, 10, and 20 μM arsenite, even at doses < 5 μM at which arsenite alone failed to upregulate Hsp70 (Fig. 5). Thus, indeed, carvacrol enhanced the stress response in arsenite-treated RPE cells, but failed to induce Hsp70 expression in the absence of a stressor. These data show that carvacrol functions as a co-inducer of Hsp70 expression in the canine RPE cell culture, which is in line with previous results in mouse and human cells (Burt et al. 2007; Wieten et al. 2010b).

**Fig. 6:**
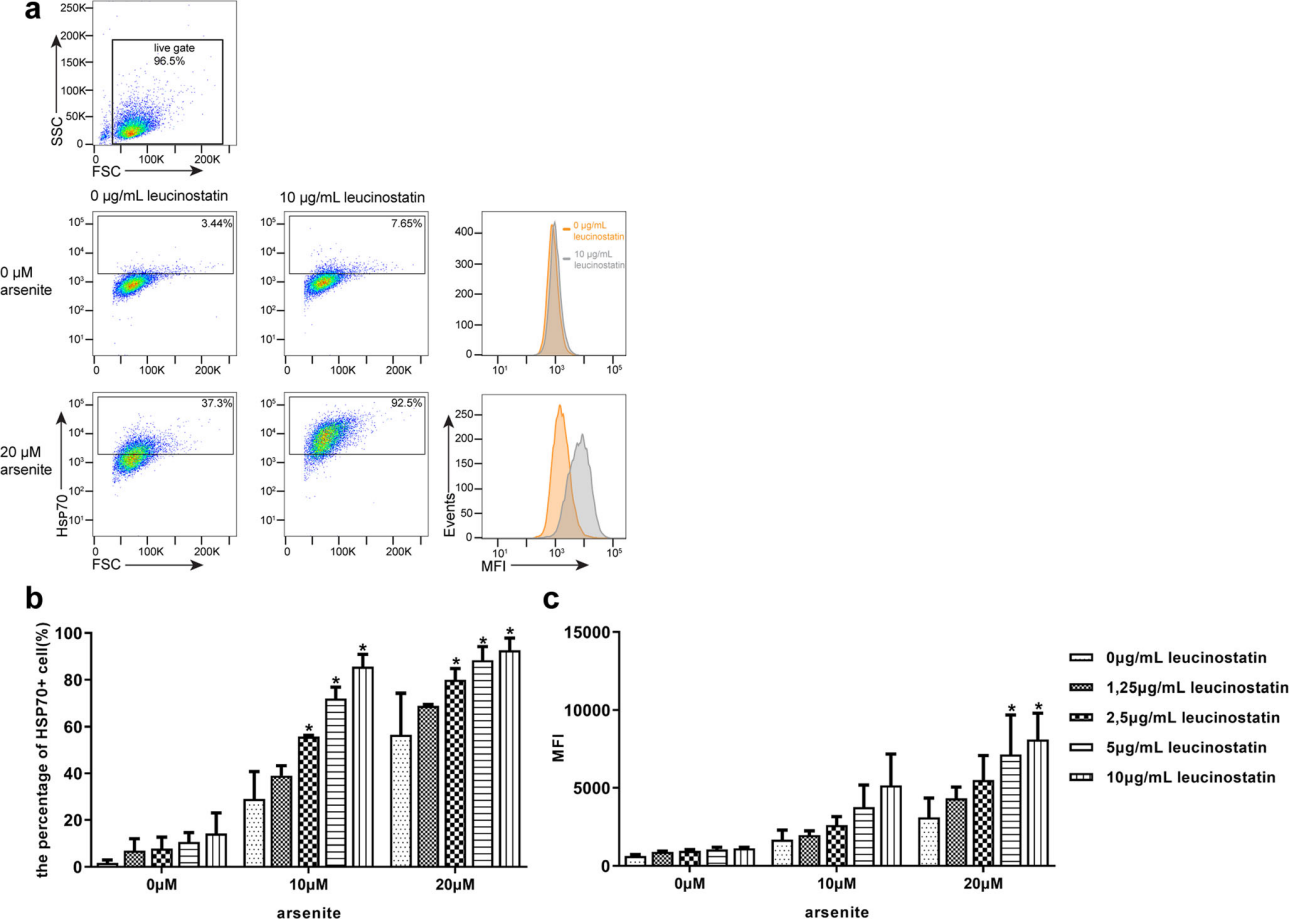
Leucinostatin co-induced Hsp70 expression in dog primary RPE cells (passages 2–4). RPE cells were incubated with the indicated concentrations of leucinostatin for 2 h at 37 °C, followed by incubation with sodium arsenite at indicated concentrations. After 16 h, Hsp70 expression in RPE cells was detected by immunofluorescence staining and flow cytometry. (a) Gating strategy and representative FACS plots, showing Hsp70 expression in sodium arsenite- and/or leucinostatin-treated RPE cells; (b) mean percentage of arsenite- and/or leucinostatin-treated RPE cells expressing Hsp70; (c) mean fluorescence intensity (MFI) of Hsp70 expression, detected in treated, cultured RPE cells. Data are representative of three independent experiments. *P* < 0.05 was considered statistically significant

Next, leucinostatin, an antimicrobial and antitumor antibiotic with potential immunomodulating capacities that is produced by the fungus *Paecilomyces lilacinus*, was tested for its ability to enhance Hsp70 expression. First, we determined Hsp70 expression in leucinostatin-treated cells in the presence or absence of arsenite by flow cytometry. As shown in Fig. 6, leucinostatin enhanced Hsp70 expression in arsenite-stressed cells in a dose-dependent fashion. Thus, these data identify leucinostatin as a new co-inducer of Hsp70 in RPE cells.

### Activation of HSF1 by leucinostatin

**Fig. 7:**
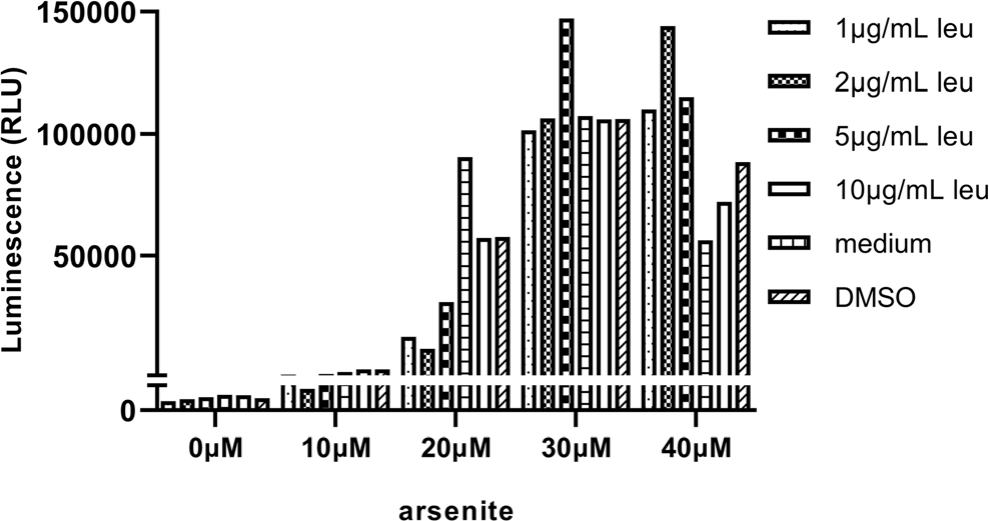
HSF1 activated by leucinostatin in DNAJB1-luc-O23 reporter cells. Stable human DNAJB1-luc-O23 cells were treated with indicated concentrations of leucinostatin and sodium arsenite at 37 °C. Luciferase activity was measured after overnight incubation

Induction of HSPs is regulated by translocated HSF1. Wieten et al. (2010b) have shown that carvacrol can enhance heat shock responses via activation of HSF1. To further examine if the HSP co-stimulatory effect of leucinostatin is mediated via HSF1, a reporter system was used (Wieten et al. 2010a). O23 cells carrying a luciferase reporter gene driven by the DNAJB1 (Hsp40) promoter were stressed by arsenite in the presence or absence of leucinostatin, as described previously for carvacrol. After 16 h, luciferase activity was measured with the Promega Steady-Glo Luciferase Assay System (Fig. 7). We found that treatment with 5 μg/ml leucinostatin in the presence of 30uM arsenite, and 1, 2, or 5 μg/ml leucinostatin in the presence 40 μM arsenite, activated transcription of the reporter from the Hsp40 promoter, which was in line with the postulated co-inducing effect. These results indicated that leucinostatin is able to promote the activation of HSF1.

## Discussion

Due to their many functions, RPE cells are a crucial cellular target during inflammation in a variety of ocular disorders. Various studies have shown that the morphology and functionality of RPE cells is changed in age-related macular degeneration (AMD) (Golestaneh et al. 2017) and in retinitis pigmentosa (Mitamura et al. 2012). Therefore, modulation and regeneration of RPE cell functionality is becoming a promising therapeutic target for multiple eye diseases. Human ophthalmologists have endeavored in upregulating Hsp70 expression in RPE cells to improve RPE health and function by sublethal thermal irradiation (Inagaki et al. 2015; Iwami et al. 2014; Wang et al. 2016), thus ameliorating ocular diseases. However, the damage threshold caused by sublethal thermal irradiation is difficult to define. Recently, compounds derived from food, herb, or approved drugs, such as carvacrol (Wieten et al. 2010b), celastrol (Westerheide et al. 2004), and geranyl geranyl acetone, (GGA) (Otaka et al. 2007) that pharmacologically elevate the level of Hsp70 expression have gained more attention. In our study, we found that leucinostatin enhanced Hsp70 expression in arsenite-stressed canine RPE cells in a dose-dependent fashion. Furthermore, leucinostatin increased the transcriptional activation of a heat shock promoter, heat shock factor-1.

Although the isolation of RPE cells has been described decades ago, there is no published protocol for isolation and culture canine RPE cells. Here, in order to find a reliable, effective, and reproducible approach for canine RPE cell isolation, we tried several published protocols used in other species (Amirpour et al. 2014; Fernandez-Godino et al. 2016; Toops et al. 2014). In our experience, it was very hard to just peel off the RPE sheet from canine choroid, which is a crucial step in isolation. When harvesting RPE cells by digesting choroid/RPE sheets, the RPE cells will be contaminated with fibroblasts or other melanocytes (data not shown). Also, the detachment of the RPE cells by dispase took a long time, which is not good for the viability of RPE cells. Therefore, our method was based on Toops et al., in which the eye cup is incubated with 0.05% trypsin to loosen PRE cells from choroid.

Purity of human RPE primary cell cultures was determined by flow cytometric detection of the RPE cell-specific protein, RPE65 (Srivastava et al. 2013). In our study, over 90% of canine RPE cells in passage 1 expressed the cytoplasmic RPE65 protein, as shown by both flow cytometry and immunocytochemistry. Furthermore, a decreasing expression of PRE65 and decreasing pigment production was seen in the following passages in longitudinal culture, which is in line with studies of human and bovine RPE cells (Alge et al. 2003; Hunt and Davis 1990; Liggett et al. 2009; Tamiya et al. 2010). In our cultures, beginning at passage 4, primary canine RPE cells underwent a phenotypic change, from epithelial to mesenchymal morphology. Higher seeding densities of canine RPE cells contributed to maintenance of hexagonal morphology, RPE65 expression, and pigment production (data not shown).

Therapeutic enhancement of HSP expression may facilitate refolding of proteins in cell stress situations and restore cellular stress resistance (Georgopoulos and Welch 1993). In addition, such enhanced expression may lead to modulation of the immune response due to induction of HSP specific regulatory T cells (Van Eden et al. 2005). Interestingly, different studies show that enhanced expression of Hsp70 in the eye not only normalizes the physiology of RPE cells (Kitamei et al. 2007), but also modulates the T cell immune response (ErdÖ and ErdÖ 1998), suggesting the therapeutic potential of Hsp70 (co-)induction in curing ocular diseases. In our study, we chose arsenite as a stressor and we determined if arsenite was capable of inducing Hsp70 expression in canine RPE cells. We found that in vitro arsenite stress induced a time- and dose-dependent increase in expression of Hsp70 in canine RPE cells, which is in line with our previous work (Wieten et al. 2010a). Interestingly, this increase of Hsp70 expression was further augmented in RPE cells by carvacrol administration which has been found to act as HSP co-inducer in various murine and human models (Wieten et al. 2010b). Using the same system, we found that leucinostatin, a candidate co-inducer, dose-dependently enhanced Hsp70 expression in arsenite-stressed cells, thus confirming that leucinostatin acts as a novel HSP co-inducer.

The expression of inducible HSPs is initiated by the nuclear translocation of HSF1. HSP inducers or co-inducers can activate HSF and induce HSPs in different ways. Otaka et al. (2007) showed that GGA could release HSF1 by binding to the C-terminus of Hsp70, resulting in induction of Hsp70; geldanamycin binds to Hsp90 to dissociate HSF1, leading to HSP induction (Zou et al. 1998); and boosting of Hsp70 by celastrol (Westerheide et al. 2004) and paeoniflorin (Hehir and Morrison 2016) is mediated by HSF1 activation. In view of this, we set out to further elucidate the HSP co-induction mechanism of leucinostatin, using the luciferase reporter O23-line. Leucinostatin appeared to be able to promote the activation of HSF1 to induce HSP expression in the O23 reporter cell line, which provides a mechanistic basis for therapeutic strategies aimed at upregulating Hsp70 expression in RPE cells.

In summary, we successfully isolated and cultured canine RPE cells, and obtained a pure primary RPE cell culture. We proved that arsenite stress induced a time and dose-dependent increase in Hsp70 expression in canine RPE cells in vitro. We investigated leucinostatin as a novel HSP co-inducer and explored its Hsp70 enhancing effects in arsenite-stressed RPE cells in a dose-dependent fashion. In addition, its Hsp70 inducing role in an HSF-1 dependent reporter system was shown. The present findings suggest that Hsp70 co-inducers like carvacrol and leucinostatin might be applied as (new) enhancers of induced Hsp70 expression, with a possible therapeutic application in inflammatory diseases of the eye.
